# Traditional Chinese Medicine Decreases the Stroke Risk of Systemic Corticosteroid Treatment in Dermatitis: A Nationwide Population-Based Study

**DOI:** 10.1155/2015/543517

**Published:** 2015-10-05

**Authors:** Kao-Sung Tsai, Chia-Sung Yen, Po-Yuan Wu, Jen-Huai Chiang, Jui-Lung Shen, Chung-Hsien Yang, Huey-Yi Chen, Yung-Hsiang Chen, Wen-Chi Chen

**Affiliations:** ^1^Institute of Chinese Medicine, School of Chinese Medicine, Graduate Institute of Integrated Medicine, School of Post-Baccalaureate Chinese Medicine, Research Center for Chinese Medicine & Acupuncture, Institute of Clinical Medical Science, College of Medicine, China Medical University, Taichung 40402, Taiwan; ^2^Departments of Dermatology, Medical Research, Obstetrics and Gynecology, and Urology, Management Office for Health Data, China Medical University Hospital, Taichung 40447, Taiwan; ^3^Department of Applied Cosmetology, Master Program of Cosmetic Science, and Department of Cultural and Creative Industries, Hungkuang University, Taichung 43302, Taiwan; ^4^Center for General Education, Feng Chia University, Taichung 40724, Taiwan; ^5^Department of Dermatology, Taichung Veterans General Hospital, Taichung 40705, Taiwan; ^6^Department of Psychology, College of Medical and Health Science, Asia University, Taichung 41354, Taiwan

## Abstract

Epidemiological studies have shown a strong association between dermatitis and stroke. Systemic corticosteroid, the mainstay treatment for dermatitis, could enhance the atherosclerotic process. Traditional Chinese Medicine (TCM) has been used for dermatitis to decrease the side effects of corticosteroid. However, the different stroke risk in dermatitis patients treated with systemic corticosteroid or TCM remains unclear. This study identified 235,220 dermatitis patients and same comorbidity matched subjects between 2000 and 2009 from database of NHRI in Taiwan. The two cohorts were followed until December 31, 2011. The primary outcome of interest was new diagnosis of stroke. The crude hazard ratio (HR) for future stroke among dermatitis patients treated with systemic corticosteroid was 1.40 (95% CI, 1.34–1.45; *P* < 0.0001) and TCM was 1.09 (95% CI, 1.05–1.13; *P* < 0.0001). The log-rank test showed a higher cumulative incidence of ischemic stroke in the patient treated with only systemic corticosteroid group than that treated with systemic corticosteroid and TCM, only TCM, and neither systemic corticosteroid nor TCM in the matched cohort during the follow-up period (*P* < 0.0001). We demonstrated that patients treated with systemic corticosteroid had an increased risk of stroke and that the risk probably decreased by TCM treatment.

## 1. Introduction

Many complementary and alternative medicine (CAM) practices have emphasized health promotion; however, this has not been the focus of the bulk of CAM research. CAM practitioners could be seen as a public health resource to increase the population's access to certain clinical preventive services [1, 2]. Eczematous dermatoses account for a large proportion of all skin disease. Some studies have suggested that dermatitis is an allergic disease in which systemic inflammation involves more than just the skin [3–5]. More evidences have shown that systemic inflammation can accelerate the progression of atherosclerosis and thrombosis with resulting ischemic stroke [6]. Epidemiological studies have shown a strong association between systemic inflammatory disease, particularly dermatoses, and cardiovascular diseases [7]. Furthermore, Su et al. demonstrated that atopic dermatitis, a chronically relapsing and constitutive skin disease, may be an independent risk factor for ischemic stroke [8].

Contemporary medicines often used combinations of topical steroid agents, systemic antihistamine, corticosteroids, and immune-modulating agents to control this frustrating disease. The treatment of dermatitis, especially systemic corticosteroid therapy, can influence the atherosclerotic process. It is believed that this treatment is atherogenic for the long-term used, partially due to effects on plasma lipoproteins, elevation of total cholesterol and triglycerides and for promoting an abnormal distribution of high-density lipoprotein subclasses [9]. The systemic corticosteroid can also indirectly accelerate the process by augmenting other traditional risk factors, including hypertension and obesity [10]. On the other hand, inflammation is associated with atherosclerosis, and therefore corticosteroid therapy could have a protective effect. Previous studies published in the literature about this issue were contradictory. The role of treatment with systemic corticosteroid or alterative treatment in the evolution of stroke in dermatitis need to be further investigated.

The decision to use CAM is multifactorial, including dissatisfaction with conventional treatment and frustration with the chronic nature course of eczema. For avoiding the potential adverse effects of systemic conventional dermatitis treatments and also to attain better clinical outcomes, many patients and practitioners have tried to seek alternative treatment [11]. Regarding the benefits, there is a raising trend of CAM treatment and the use of CAM is actually associated with eczema prevalence [12]. Traditional Chinese Medicine (TCM) is one of the popular alternative treatments for dermatitis in Asia and the world [13, 14]. The aim of this study was to determine the different risk of stroke in dermatitis patients treated with systemic corticosteroid or TCM by using a nationwide database and proved a part of a structured initiative to established evidenced-based clinical recommendation for management of comorbidities in dermatitis.

## 2. Materials and Methods

### 2.1. Data Sources

Taiwan's National Health Insurance (NHI) program, implemented by the government in March 1995, provides comprehensive health care to almost all Taiwanese citizens, with a coverage rate of more than 99% of Taiwan's entire population, and contracted with 97% of hospitals and 92% of clinics. The National Health Research Institute (NHRI) of Taiwan manages and publicly releases for research purposes multiple NHI databases that include information about basic patient characteristics, date of visit, diagnoses codes for the International Classification of Diseases, Ninth Revision, Clinical Modification (ICD-9-CM) codes, detailed claims data for examinations, disease management, and drug prescriptions for all admitted patients and outpatients [15, 16]. The NHRI created research data sets including a random sample of 1,000,000 subjects from the registry of all NHI enrollees in 2000, with the encryption of personal information that could identify any individual patient. We obtained these data sets of NHRI from 2000 to 2011 for use as our research database. This study was approved by the Institutional Review Board of CMU-REC-101-012 from Institutional Review Board approval of Public Health, Social and Behavioral Science Committee Research Ethics Committee, China Medical University and Hospital.

### 2.2. Study Design and Population

This population-based cohort study utilizing a nationwide database was conducted of two groups. The population with dermatitis (aged ≥ 20 years) were identified by codes 690.X, 691.X, and 692.X in the ICD-9-CM and newly dermatitis diagnosis (at least two medical visits) between 1 January 2000 and 31 December 2009 and followed up until December 31, 2011. Subjects who have a past history of stroke before the enrollment date were excluded from the study group. Systemic corticosteroid or TCM coding was obtained for medication variant control in advanced step of analysis. We included the most common prescribed systemic corticosteroids: dexamethasone, methylprednisolone, and prednisolone. Treatment was divided into non-TCM and nonsystemic steroid user, only TCM user, only systemic steroid user, and TCM and systemic steroid user. The primary outcome of interest was new diagnosis of stroke (ICD-9-CM codes: 430–438). For stroke type analysis, we separated hemorrhagic stroke (ICD-9-CM codes 430, 431, and 432) and compared the ischemic stroke (ICD-9-CM codes 433–438) in further adjusted hazard ratio analysis. The date for dermatitis diagnosis was defined as index date. All the subjects were followed from the index date to occurrence of endpoint or until December 31, 2011, whichever was first, and the observations on the last dates were considered as censored observations.

### 2.3. Comparison Group

Subjects without dermatitis were randomly selected from the same data set. Each patient with newly diagnosed dermatitis in the NHRI database was pair-matched with one subject of the same age, sex, and index year. TCM or systemic corticosteroid medications and comorbidities (allergic rhinitis, asthma, urticarial, diabetes mellitus, hypertension, hyperlipidemia, and atrial fibrillation) were not matched. We selected comparison subjects using incidence density sampling by computer programming [17]. In the comparison group, subjects who have past history of stroke before enrollment were also excluded as the study group.

To determine stroke and survival analyses adjusting for age, sex, comorbidities, and medications were carried out with Cox's proportional hazards model. All enrollees were followed from the date of enrollment until the first diagnosis of stroke or censored date of death, or date of withdrawal from the insurance, or until 31 December 2011.

### 2.4. Potential Confounders

In the analysis of the effect of different treatment, systemic corticosteroid or TCM, in patients with dermatitis on the outcome of stroke, we controlled the age and sex and identified the following comorbidities as potential confounders: diabetes mellitus (ICD-9-CM code: 250), hypertension (ICD-9-CM codes: 401–405), hyperlipidemia (ICD-9-CM codes: 272.0, 272.1, 272.2, 272.3, and 272.4), and atrial fibrillation (ICD-9-CM code: 427.31).

### 2.5. Statistical Analysis

Person-years of two populations were calculated from baseline to the occurrence of stroke or closing date (December 31, 2011). All statistical analyses were performed using SAS version 9.4 software (SAS Institute, Inc., Cary, NC).

All data are expressed as mean standard deviation or *n* (%) unless otherwise stated. Comparisons between groups were performed using Student's *t*-test for continuous variables and Pearson's chi-square test, as appropriate, for categorical variables. The Cox's proportional hazards model was used to estimate the hazards ratio for the progression of outcome. The probability of survival difference between each group with dermatitis user and nondermatitis users was tested with the log-rank test. The Kaplan-Meier method was used to plot the cumulative incidence. Cox proportional hazard model was used to calculate the hazard ratios and 95% confidence interval of stroke for patients with dermatitis compared with nondermatitis user. All analyses were carried out with SAS statistical software. All statistical tests were performed at the two-tailed significance level of 0.05. A *P* value < 0.05 was considered statistically significant.

## 3. Results

Clinical characteristics of this study population identified patients newly diagnosed with dermatitis between 1 January 2000 and 31 December 2009. After excluding patients aged under 20 years or with antecedent stroke, 235,220 patients with dermatitis were included in the analyses. Other 235,220 patients without dermatitis were selected by 1 : 1 matching by age, sex, and index year. The study subjects were predominantly female (58.13%), and the median age was 41.9 ± 15.5 years for dermatitis cohort group and 41.5 ± 15.9 years for nondermatitis cohort group.  Table 1 shows that basic characteristics and selected comorbidities were similar between groups.

**Figure 1:**
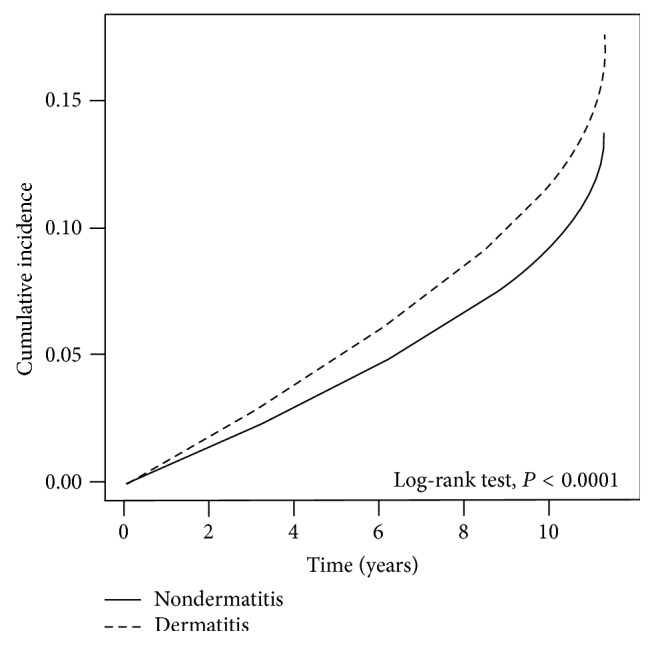
The estimated cumulative incidence of stroke between the dermatitis cohort and the nondermatitis cohort by Kaplan-Meier analysis.

Predictors of difference stroke risk between systemic corticosteroid and TCM treatment in patients with dermatitis were conducted in this study. During the follow-up period, 206,402 (87.75%) patients with dermatitis were treated with systemic corticosteroid and 160,541 (68.25%) were comparison subjects. 207,890 (88.38%) patients with dermatitis treated with TCM and 183,949 (78.20%) were comparison subjects. Also, subject with and without dermatitis had 78.47 and 57.22 percentage who had used both TCM and systemic steroid. We also found that 13,079 (5.65%) patients with dermatitis and 10,006 (4.25%) comparison subjects experienced stroke attack. Analyzing different stroke type, 12,450 (5.29%) patients with dermatitis and 9,277 (3.94%) comparison subjects had Ischemic stroke attack. However, there was no statistically difference in patients with dermatitis and comparison subjects that experienced hemorrhagic stroke attack. The log-rank test showed a higher cumulative incidence of stroke in the dermatitis group than in the matched cohort during the follow-up period (*P* < 0.0001, Figure 1), suggesting that patients with dermatitis had an increased risk of stroke in the long term.

**Figure 2:**
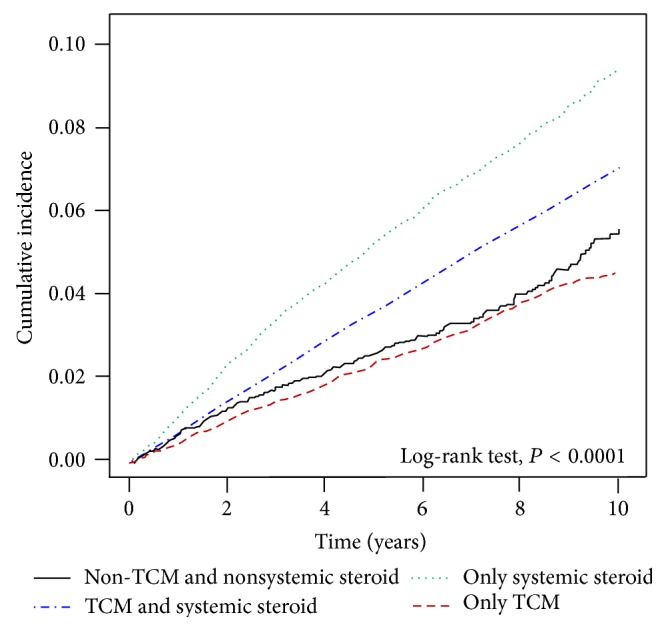
The estimated cumulative incidence of ischemic stroke between those treated with CTM or systemic corticosteroid in the patients of dermatitis cohort by Kaplan-Meier analysis.

After adjusting for age, gender, comorbidities, and medications, we compared with comparison subjects and stratified Cox's proportional hazard regression demonstrated that the crude hazard ratio (HR) for future stroke among patients with dermatitis was 1.13 (95% confidence interval, (95% CI, 1.1–1.16; *P* < 0.0001) and ischemic stroke among patients with dermatitis was 1.16 (95% CI, 1.12–1.19; *P* < 0.0001). Furthermore, compared with non-TCM and nonsystemic steroid user, the adjusted hazard ratio (HR) for future stroke among patients treated with only TCM was 1.22 (95% CI, 1.13–1.32; *P* < 0.0001), only systemic steroid was 1.55 (95% CI, 1.43–1.67; *P* < 0.0001) and TCM and systemic steroid user was 1.64 (95% CI, 1.53–1.76) (Tables 2 and 3). These HR results suggested that dermatitis and patient treated with systemic corticosteroid or TCM may be an independent risk factor for stroke. The log-rank test showed a higher cumulative incidence of ischemic stroke in the patient with dermatitis and treated with systemic corticosteroid group than treated with systemic corticosteroid and TCM, only TCM, and neither systemic corticosteroid nor TCM in the matched cohort during the follow-up period (*P* < 0.0001, Figure 2).

We also identified the following independent factors determining the risk of future stroke. The adjusted HRs of stroke were significantly lower in female than male (HR: 0.81; 95% CI, 0.78–0.83; *P* < 0.0001) and increased with increasing age. Significant adjusted HRs of stroke in Cox proportional hazard models were asthma (HR: 1.07; 95% CI, 1.02–1.12; *P* = 0.0073), diabetes mellitus (HR: 1.37; 95% CI, 1.32–1.41; *P* < 0.0001), hypertension (HR: 1.87; 95% CI, 1.81–1.93; *P* < 0.0001), and atrial fibrillation (HR: 1.70; 95% CI, 1.51–1.91; *P* < 0.0001).

## 4. Discussion

In this large population-based cohort study, we demonstrated that the patient with dermatitis treated with systemic corticosteroid is a risk factor for stroke and the patients treated with TCM may decrease incidence of this risk. Patients with dermatitis treated with TCM had decreased incidence of ischemic stroke compared with the corticosteroid group. These findings support the concept that dermatitis may exert a systemic effect contributing to stroke and different treatments are important confounders.

Corticosteroid is the mainstay treatment for dermatitis, with the route of administration and dosage schedule dependent primarily on the severity, while complications range in severity and frequency, which are generally considered to be depend on the dosage and/or duration of corticosteroid. However, the adverse effects result from not only the cumulative corticosteroid dose but also high-dose corticosteroid treatment which was significantly associated with development of stroke [18]. Obesity, diabetes mellitus, hypertension, atrial fibrillation, and hyperlipidemia may be worse as corticosteroid treated; furthermore, these adverse effects may contribute to the later development of atherosclerosis and ischemic stroke [19–21].

The atherosclerotic changes or stroke are associated with inflammatory processes resulting from several dermatoses, such as atopic dermatitis [8], dermatitis herpetiformis [22], systemic lupus erythematosus [23], bullous pemphigoid [24], drug rash eosinophilia and systemic symptoms (DRESS) [25], and psoriasis [26]. Possible explanations for the high risk of stroke in patients with dermatitis are atherosclerotic changes [4, 14, 23], oxidative stress [27], and activation of the coagulation system related to chronic inflammation [28, 29]. Increasing evidence has shown that systemic inflammation can promote the progression of atherosclerosis and thrombosis to ischemic stroke [6]. There are several possible mechanisms of dermatitis resulting in stroke. First, the elevation of platelet activation and reducing fibrinolysis were founded in patients with chronic inflammatory allergic diseases such as atopic dermatitis [30, 31]. Second, mast cell may participate in atherosclerosis by releasing proinflammatory cytokines, chemokines, and proteases to induced inflammatory cell recruitment, cell apoptosis, and angiogenesis [32, 33]. Third, increased serum IgE levels in myocardial infarction patients and mast cell accumulated in atherosclerotic lesions [34]. Fourth, hypereosinophilia may play an import role in some of these dermatoses, included dermatitis, bullous pemphigoid, and DRESS. Thrombosis may be related to eosinophilic hypothiocyanous acid productions, which lead to a prothrombotic state [35]. Furthermore, encephalopathy may arise from small cerebral stroke or direct eosinophil toxicity [36]. Dermatitis is an allergic disease, like asthma; it probably exerts systemic inflammatory effect in a similar fashion, thereby contributing to cardiovascular or cerebrovascular consequences. However, allergic rhinitis and urticarial seem to be of milder and less systemic inflammation than other atopic diseases.

A number of studies on TCM have been performed, with a collective result of symptom improvement and decreased levels of inflammatory cytokines. Since standard TCM prescribed of many herbs combined in different forms and dosed differently depending on each individual patient, randomized control trials in this area have been difficult to perform. It has been postulated that Zemaphyte might work as an efficient antioxidant, capable of scavenging both superoxide and hydroxyl and preventing peroxidation of biological membranes. Pentaherbs formulation, another TCM prescription formula, postulated that suppression of the low-affinity receptors for IgE on antigen-presenting cell, modulated mast cells and inhibited the inflammatory mediators from mast cells [37], and possessed immunomodulatory effects and inflammatory mediators [38]. Methodological advantages of the interdisciplinary secondary database utilized include a high degree of generalizability, completeness and absence of recall bias due to prospective input of diagnoses and research questions and large sample size [39]. In this study, we replicated the previous reported positive association between the major stroke risk factors and found that atrial fibrillation, hypertension, diabetes, and hyperlipidemia seem to be very sensitive to change to multivariate models (Table 2). This may be due to the low prevalence of those traditional risk factors for stroke in the dermatitis group. We demonstrated that dermatitis may be an independent risk factor for ischemic stroke. In light of our limited understanding of the exact mechanisms explaining the adverse stroke risk factors in dermatitis patients, it has been speculated that the established association between stroke and different treatments might explain these findings.

Our study has several limitations. First, patients with dermatitis and stroke were identified using a diagnostic code in a database, introducing the possibility of misclassification because of coding errors or misdiagnosis. Second, some potential risk factors, including obesity, smoking, alcohol use, and family history of cardiovascular disease, were not included in our analyses because these data were not available. Third, the follow-up period may not have been sufficiently long to detect stroke development because atopic dermatitis always course in child to teenager but stroke often attacks Middle-aged to elderly patients. Fourth, we could not directly evaluate the severity of dermatitis stroke, the accumulated dosage of systemic corticosteroid, ingredients of TCM, and each comorbidity. Finally, because we did not have the information of causes of death, stroke may be a cause of death but was not recorded as an endpoint. The role of inflammation biomarkers, ingredients of TCM, and the relationship between TCM, dermatitis, and stroke are not clear. Further research is needed to determine the possible pathogenic mechanisms of TCM prescribed in dermatitis and stroke which is necessary.

## 5. Conclusions

This large population-based study demonstrated that patients treated with systemic corticosteroid had an increased risk of stroke and that the risk probably decreased by TCM treatment.

## Figures and Tables

**Table 1:** Demographic characteristics and comorbidity in patients with and without dermatitis.

Variables	Dermatitis				*P* value^†^
	No		Yes		
	(*N* = 235220)		(*N* = 235220)		
	*n*	%	*n*	%	
Sex					
Female	136734	58.13	136734	58.13	0.99
Male	98486	41.87	98486	41.87	
Age, years					
20–39	118623	50.43	118623	50.43	0.99
40–49	51025	21.69	51025	21.69	
50–59	31596	13.43	31596	13.43	
≥60	33976	14.44	33976	14.44	
Mean (SD)^†^	41.47 ± 15.92		41.93 ± 15.52		<0.0001
Comorbidity					
Asthma	9394	3.99	14123	6.00	<0.0001
Allergic rhinitis	21178	9.00	33243	14.13	<0.0001
Urticaria	11167	4.75	26983	11.47	<0.0001
Diabetes mellitus, DM	14864	6.32	20171	8.58	<0.0001
Hypertension	32216	13.70	40360	17.16	<0.0001
Hyperlipidemia	14819	6.30	21579	9.17	<0.0001
Atrial fibrillation, AF	625	0.27	700	0.30	0.0391
Treatment					
TCM (excluded acupuncture)	183949	78.20	207890	88.38	<0.0001
Systemic corticosteroid	160541	68.25	206402	87.75	<0.0001
Dexamethasone	103693	44.08	146470	62.27	<0.0001
Methyl prednisolone	35995	15.30	62122	26.41	<0.0001
Prednisolone	128167	54.49	182906	77.76	<0.0001
Treatment (new)					
Non-TCM and nonsystemic steroid	25312	10.76	5507	2.34	<0.0001
Only TCM	49367	20.99	23311	9.91	
Only systemic steroid	25959	11.04	21823	9.28	
TCM and systemic steroid	134582	57.22	184579	78.47	
Outcome					
Stroke	10006	4.25	13079	5.56	<0.0001
Hemorrhagic stroke	1543	0.66	1463	0.62	0.1432
Ischemic stroke	9277	3.94	12450	5.29	<0.0001

Chi-square test; ^†^two-sample *t*-test.

**Table 2:** Cox model measured hazard ratio and 95% confidence intervals of stroke associated with dermatitis and covariates.

Characteristics	Crude			Adjusted		
	HR	(95% CI)	*P* value	HR	(95% CI)	*P* value
Dermatitis (ref = no)						
Yes	1.27	(1.23–1.3)	<0.0001	1.13	(1.10–1.16)	<0.0001
Gender (ref = male)						
Female	0.67	(0.66–0.69)	<0.0001	0.79	(0.77–0.81)	<0.0001
Age, years (ref = 20–39)						
40–49	4.18	(3.97–4.41)	<0.0001	3.75	(3.56–3.96)	<0.0001
50–59	9.58	(9.1–10.08)	<0.0001	7.27	(6.90–7.67)	<0.0001
≥60	25.70	(24.54–26.92)	<0.0001	16.17	(15.37–17.00)	<0.0001
Comorbidity						
Asthma (ref = no)						
Yes	2.42	(2.32–2.53)	<0.0001	1.07	(1.03–1.12)	0.0019
Allergic rhinitis (ref = no)						
Yes	1.02	(0.97–1.06)	0.4694	0.95	(0.91–0.99)	0.0158
Urticaria (ref = no)						
Yes	1.12	(1.07–1.17)	<0.0001	0.99	(0.94–1.04)	0.6715
DM (ref = no)						
Yes	4.20	(4.07–4.33)	<0.0001	1.35	(1.30–1.39)	<0.0001
Hypertension (ref = no)						
Yes	6.26	(6.1–6.43)	<0.0001	1.87	(1.82–1.93)	<0.0001
Hyperlipidemia (ref = no)						
Yes	3.08	(2.97–3.18)	<0.0001	1.00	(0.96–1.04)	0.9719
AF (ref = no)						
Yes	7.04	(6.28–7.9)	<0.0001	1.68	(1.50–1.89)	<0.0001
Treatment (ref = non-TCM and nonsystemic steroid)						
Only TCM	0.98	(0.90–1.06)	0.5871	1.22	(1.13–1.32)	<0.0001
Only systemic steroid	2.19	(2.03–2.36)	<0.0001	1.55	(1.43–1.67)	<0.0001
TCM and systemic steroid	1.66	(1.55–1.77)	<0.0001	1.64	(1.53–1.76)	<0.0001

HR: hazard ratio; CI: confidence interval.

Adjusted HR: adjusted for age, gender, treatment, and comorbidity in Cox proportional hazards regression.

**Table 3:** Cox model measured hazard ratio and 95% confidence intervals of ischemic stroke associated with dermatitis and covariates.

Characteristics	Crude			Adjusted		
	HR	(95% CI)	*P* value	HR	(95% CI)	*P* value
Dermatitis (ref = no)						
Yes	1.30	(1.27–1.34)	<0.0001	1.16	(1.12–1.19)	<0.0001
Gender (ref = male)						
Female	0.69	(0.67–0.71)	<0.0001	0.81	(0.78–0.83)	<0.0001
Age, years (ref = 20–39)						
40–49	4.60	(4.34–4.87)	<0.0001	4.12	(3.89–4.37)	<0.0001
50–59	10.88	(10.3–11.5)	<0.0001	8.25	(7.8–8.73)	<0.0001
≥60	29.39	(27.95–30.89)	<0.0001	18.48	(17.5–19.5)	<0.0001
Comorbidity						
Asthma (ref = no)						
Yes	2.45	(2.35–2.57)	<0.0001	1.07	(1.02–1.12)	0.0073
Allergic rhinitis (ref = no)						
Yes	1.04	(1–1.09)	0.0829	0.97	(0.92–1.01)	0.1195
Urticaria (ref = no)						
Yes	1.12	(1.07–1.18)	<0.0001	0.98	(0.94–1.03)	0.5232
DM (ref = no)						
Yes	4.35	(4.22–4.49)	<0.0001	1.37	(1.32–1.41)	<0.0001
Hypertension (ref = no)						
Yes	6.48	(6.31–6.65)	<0.0001	1.87	(1.81–1.93)	<0.0001
Hyperlipidemia (ref = no)						
Yes	3.18	(3.07–3.3)	<0.0001	1.01	(0.97–1.05)	0.6112
AF (ref = no)						
Yes	7.24	(6.44–8.14)	<0.0001	1.70	(1.51–1.91)	<0.0001
Treatment (ref = non-TCM and nonsystemic steroid)						
Only TCM	1.01	(0.93–1.09)	0.8967	1.25	(1.15–1.36)	<0.0001
Only systemic steroid	2.23	(2.06–2.42)	<0.0001	1.56	(1.44–1.69)	<0.0001
TCM and systemic steroid	1.72	(1.60–1.85)	<0.0001	1.68	(1.57–1.81)	<0.0001

HR: hazard ratio; CI: confidence interval.

Adjusted HR: adjusted for age, gender, treatment, and comorbidity in Cox proportional hazards regression.
